# Which primary care practitioners have poor human papillomavirus (HPV) knowledge? A step towards informing the development of professional education initiatives

**DOI:** 10.1371/journal.pone.0208482

**Published:** 2018-12-13

**Authors:** Lisa A. McSherry, Eamonn O’Leary, Stephan U. Dombrowski, Jill J. Francis, Cara M. Martin, John J. O’Leary, Linda Sharp, on behalf of the ATHENS (A Trial of HPV Education and Support) Group

**Affiliations:** 1 National Cancer Registry, Cork, Ireland; 2 Division of Psychology, University of Stirling, Stirling, Scotland; 3 School of Health Sciences, City University London, London, England; 4 Coombe Women and Infants University Hospital, Dublin, Ireland; 5 Institute of Health & Society, Newcastle University, Newcastle-upon-Tyne, England; The University of Sydney, AUSTRALIA

## Abstract

**Background:**

Primary care practitioners (PCP) play key roles in cervical cancer prevention. Human papillomavirus (HPV) knowledge is an important influence on PCPs’ cervical cancer prevention-related behaviours. We investigated HPV knowledge, and associated factors, among general practitioners (GPs) and practice nurses.

**Methods:**

A survey, including factual questions about HPV infection and vaccination, was mailed to GPs and practice nurses in Ireland. Multivariable logistic regression was used to determine which PCPs had low knowledge (questions correctly answered: infection ≤5/11; vaccination: ≤4/10). Questions least often answered correctly were identified.

**Results:**

697 PCPs participated. For HPV infection, GPs and practice nurses answered a median of nine and seven questions correctly, respectively (p<0.001). Significantly associated with low HPV infection knowledge were: being a practice nurse/male GP; working fewer hours/week; not having public patients; and having never taken a cervical smear. For HPV vaccination, both GPs and practice nurses answered a median of six questions correctly (p = 0.248). Significantly associated with low HPV vaccination knowledge were: being a practice nurse/male GP; working more years in general practice, fewer hours/week, in a smaller practice or in a practice not specialising in women’s health; and having never taken a smear. Six HPV infection questions, and seven HPV vaccination questions, were not answered correctly by >⅓ of PCPs.

**Conclusions:**

There are important limitations in HPV infection and vaccination knowledge among PCPs. By identifying factors associated with poor knowledge, and areas of particular uncertainty, these results can inform development of professional education initiatives thereby ensuring women have access to uniformly high-quality HPV-related information and advice.

## Introduction

The causal relationship between cervical cancer and “high-risk” human papillomavirus (HPV) infection is reshaping cervical cancer prevention strategies internationally [1]. Primary care practitioners (PCPs), both doctors and nurses, play key roles in cervical cancer prevention in many countries. In the UK and Ireland, for example, they take most cervical screening tests. Traditionally this has involved conducting cervical cytology tests, but some countries have moved to, and others intend to implement, screening based on HPV testing, with the HPV test provided to women in the same way as a cytology test. In addition, PCPs may also advise patients about HPV infection or vaccination and/or provide HPV vaccination [2,3].

PCPs can significantly impact women’s cervical cancer prevention behaviours. They may positively or negatively influence women’s screening participation decisions and decision-making around HPV vaccination [4–6]. For example, US studies consistently show that health professional recommendation is one of the strongest influences on parental decisions regarding HPV vaccination of their children [7,8]. However, in theoretically-robust research, we found that lack of HPV knowledge among PCPs was a key barrier to them performing cervical cancer prevention-related behaviours appropriately [2]. Other studies indicate that practitioners with greater knowledge are more likely to discuss or recommend HPV vaccination [9–13]. Furthermore, in a recent US study, HPV vaccine initiation and completion rates among adolescents were higher in the practice populations of primary care physicians with greater HPV infection and vaccination knowledge [14]. Therefore, good knowledge appears a prerequisite for optimal cervical cancer prevention-related behaviours among PCPs.

The success of the new prevention strategies also requires that women (and the wider population) are adequately informed about HPV, understand new protocols/programmes, and find them acceptable. However, public awareness of HPV is poor, understanding is limited, and misconceptions are common [15–18]. Women regard PCPs as trusted sources of HPV-related information and support [19–21]. Knowledgeable PCPs are, therefore, essential to effectively meet people’s HPV information and advice needs.

Previous research suggests some PCPs have significant gaps in their HPV-related knowledge [22–28]. However, most evidence is from North America and there has been very little investigation of primary care nurses’ knowledge. We investigated HPV knowledge among primary care doctors and nurses; determined which PCPs have poor knowledge; and identified particular aspects of HPV knowledge about which PCPs are most uncertain. Specifically, we set out to compare whether knowledge levels of GPs and practice nurses vary, and whether male and female GPs have different levels of knowledge; some tentative indications of this emerged in our previous qualitative work [2]. Such information is essential to inform development and targeting of professional education strategies, and ensure women receive the most appropriate advice and support.

## Methods

### Setting

The study setting was Ireland, which has a mixed public-private healthcare system. Citizens pay for primary care services unless they possess a medical card, eligibility for which is based on means and age. General practitioners (GPs) may choose whether to include medical card holders (“public patients”) on their patient list. The cervical screening programme, CervicalCheck, began national roll-out in 2008 and provides women aged 25–60 with free cytology tests every 3–5 years [29]. Women may also obtain cytology tests privately from a GP or Well Woman Centre. At the time of the fieldwork, HPV testing was available privately in primary care. HPV vaccinations have been available privately in primary care since 2006 and a publicly-funded schools-based vaccination programme for girls aged 12 commenced in 2010 [30].

### Design

During May-August 2011, we conducted a cross-sectional postal survey of GPs and practice nurses.

### Study population

In total 1760 practitioners (880 GPs and 880 practice nurses) were sampled to be invited to take part in the survey. The GP sampling frame was the Irish Medical Directory, which is Ireland’s most comprehensive listing of healthcare professionals. Simple random sampling was used to select the sample. Since there is no national practice nurse register, half of the nurse sample was selected, using simple random sampling, from a list of members of the Irish Practice Nurses Association and half from practice nurses registered with CervicalCheck. All of the individuals on both nurse sampling frames were female.

### Statistical power

Sample size was based on statistical power to detect, as significant, associations between a personal or practice-related characteristic or cervical screening-related behaviour and risk of low HPV knowledge. “Low” knowledge was defined as the lowest quintile of respondents; we aimed for ≥100 practitioners in this group. Assuming the characteristic had a prevalence of 25%, 100 respondents with a “low” score and 400 with a “higher” score would have 82% power to detect, as statistically significant, an odds ratio of 2.0 (alpha = 0.05, two-sided test). Based on assumption that the response rate would be 30% (consistent with other PCP surveys in Ireland), and allowing for some practitioners not being contactable, we decided to sample 1760 practitioners, evenly split between GPs and practice nurses.

### Questionnaire

The questionnaire was 12 pages long, including the front and back cover and the embedded consent form; a copy is available from the corresponding author on request. Content was theoretically informed based on findings from our qualitative work [2]. The sections were ordered as follows: practitioners’ cervical screening behaviours; HPV infection-related attitudes, beliefs, behaviours and knowledge; HPV vaccination-related attitudes, beliefs, behaviours and knowledge; HPV testing-related attitudes and beliefs; three clinical scenarios regarding HPV; and practitioners’ personal and practice characteristics. HPV infection knowledge was assessed using 11 factual statements based on Jain et al [20]. Ten factual statements about HPV vaccination were developed using evidence-based information from sources including the European Cervical Cancer Association (ECCA) and the National Immunisation Office Ireland. Response options for each statement were agree, disagree and unsure.

### Data collection

We used several strategies that have been shown to increase response rates to postal surveys [31]. Subjects were sent a personalised pre-contact letter stating that they would shortly receive a survey. A few indicated that they did not wish to receive the survey; for a few others, letters were returned undelivered or marked that the recipient no longer practiced. These practitioners were removed and the survey was mailed, with a personalised cover letter, to 876 GPs and 866 practice nurses. The questionnaire and cover letter indicated that respondents would be entered into a prize draw to win a high-street voucher. Non-respondents were sent a postal reminder after two weeks followed, if required, by a telephone call two weeks later.

### Ethics

Ethical approval was provided by the Irish College of General Practitioners (ICGP). The ICGP Ethics Committee has responsibility for reviewing and approving research in primary care in Ireland.

### Statistical analysis

Practitioners were included in the analysis if they had answered at least half of the HPV infection or HPV vaccination factual questions. Two knowledge scores, one for infection and one for vaccination, were generated for each participant comprising the number of correctly answered questions. Correct/incorrect answers were defined based on what was known about HPV infection and vaccination at the time of the fieldwork. Hence, if an individual answered 8 of the 11 HPV infection questions, and their responses to 5 of these were correct, their infection knowledge score was 5 out of a possible 11. Similarly if they answered all 11 HPV infection questions and responses to 5 were correct, their knowledge score was 5/11.

Respondents’ personal and practice characteristics were summarised in terms of numbers and percentages or means (with standard deviations) overall and for GPs and practice nurses separately. The knowledge score distributions were skewed so we summarised average scores for GPs and practice nurses in terms of medians and compared GPs’ and practice nurses’ knowledge score distributions using the non-parametric Wilcoxson rank-sum test. Practitioners with scores in the lowest quintile of the overall distribution were designated as having “low” knowledge. Multivariable logistic regression was used to identify factors associated with low knowledge, separately for infection and vaccination. Practitioners’ personal and practice characteristics and cervical screening behaviours (Table 1) were considered for inclusion in the models. Those significant on likelihood ratio tests (p<0.05) were retained in the final models. Following this, responses to individual questions were classified as “correct” or “incorrect”; the latter group included those who provided wrong answers, indicated they were unsure of the answer or declined to answer. Using chi-square tests, we tested for associations between correct/incorrect answers and practitioner group (female GPs/male GPs/practice nurses). A secondary analysis distinguished between wrong, unsure and missing responses.

**Table 1:** Personal and practice characteristics, and cervical screening related behaviours, of PCPs who participated in the survey, numbers and percentages (or means and standard deviation)^1^.

		All PCPs		GPs		Practice Nurses	
***Total***		**697**	***100*.*0%***	**238**	**34.1%**	**459**	**65.9%**
**Personal characteristics**							
Sex	Female	604	*86*.*7%*	145	*60*.*9%*	459	*100*.*0%*
	Male	93	*13*.*3%*	93	*39*.*1%*	0	*0*.*0%*
Number of hours worked per week: mean (sd)		29.1 (12.2)		37.6 (14.1)		25.0 (8.7)	
Number of years in general practice	<10 years	298	*43*.*4%*	32	*13*.*9%*	266	*58*.*3%*
	10–19 years	244	*35*.*6%*	78	*33*.*9%*	166	*36*.*4%*
	20–39 years	135	*19*.*7%*	112	*48*.*7%*	23	*5*.*0%*
	40+ years	9	*1*.*3%*	8	*3*.*5%*	1	*0*.*2%*
Ever worked or trained outside Ireland	Yes	366	*53*.*6%*	118	*51*.*1%*	248	*54*.*9%*
	No	317	*46*.*4%*	113	*48*.*9%*	204	*45*.*1%*
***Practice characteristics***							
Practice location	Dublin city	141	*20*.*7%*	52	*22*.*7%*	89	*19*.*6%*
	A city other than Dublin	76	*11*.*1%*	24	*10*.*5%*	52	*11*.*5%*
	A town	316	*46*.*3%*	102	*44*.*5%*	214	*47*.*2%*
	A village	124	*18*.*2%*	44	*19*.*2%*	80	*17*.*7%*
	Other country	25	*3*.*7%*	7	*3*.*1%*	18	*4*.*0%*
Solo GP practice	Yes	118	*17*.*3%*	42	*18*.*4%*	76	*16*.*7%*
	No	566	*82*.*7%*	186	*81*.*6%*	380	*83*.*3%*
Number of GPs in practice: mean (sd)		3.3 (2.1)		3.2 (2.2)		3.3 (2.1)	
Any female GP(s)in practice	Yes	559	*81*.*7%*	200	*87*.*7%*	359	*78*.*7%*
	No	125	*18*.*3%*	28	*12*.*3%*	97	*21*.*3%*
Any practice nurse(s) in practice^2^	Yes	181	*79*.*4%*	181	*79*.*4%*	-	
	No	47	*20*.*6%*	47	*20*.*6%*	-	
Practice has public patient list^3^	Yes	662	*95*.*9%*	214	*92*.*6%*	448	*97*.*6%*
	No	28	*4*.*1%*	17	*7*.*4%*	11	*2*.*4%*
Practice specialises in women’s health	Yes	448	*65*.*6%*	155	*66*.*8%*	293	*65*.*0%*
	No	235	*34*.*4%*	77	*33*.*2%*	158	*35*.*0%*
***Cervical screening-related behaviours***							
Registered smeartaker^4^	Yes	651	*94*.*2%*	212	*91*.*4%*	439	*95*.*6%*
	No	40	*5*.*8%*	20	*8*.*6%*	20	*4*.*4%*
Number of cytology tests taken now compared to three years ago	I take about the same	87	*12*.*9%*	47	*20*.*6%*	40	*8*.*9%*
	I take fewer	91	*13*.*5%*	81	*35*.*5%*	10	*2*.*2%*
	I take more	471	*69*.*7%*	89	*39*.*0%*	382	*85*.*3%*
	I have never taken a smear	27	*4*.*0%*	11	*4*.*8%*	16	*3*.*6%*
Attended smeartaking seminar in past year	Yes	327	*47*.*9%*	61	*26*.*8%*	266	*58*.*5%*
	No	356	*52*.*1%*	167	*73*.*2%*	189	*41*.*5%*
Attended HPV seminar in past year	Yes	106	*16*.*4%*	30	*13*.*5%*	76	*17*.*9%*
	No	540	*83*.*6%*	192	*86*.*5%*	348	*82*.*1%*

^1^ subjects with missing values for each variable not shown so numbers may not sum to 697; % are of those who responded to the question

^2^ questions relevant to GPs only

^3^ i.e. patients with medical cards, which provide free primary care services and subsidized prescription medicines

^4^ registered with CervicalCheck, the national cervical cancer screening programme

## Results

697 PCPs participated (response rate 40%; GPs: 238 (27% response); practice nurses: 459 (53%)). Table 1 shows practitioners’ personal and practice characteristics and cervical screening behaviours. All participating practice nurses, and 61% of GPs, were female.

### HPV infection

#### Knowledge score distribution

Of the 690 practitioners who answered at least half the questions, there were four (0.6%) who either answered zero or one question correctly, and 28 (4%) who answered all 11 questions correctly.

**Fig 1:**
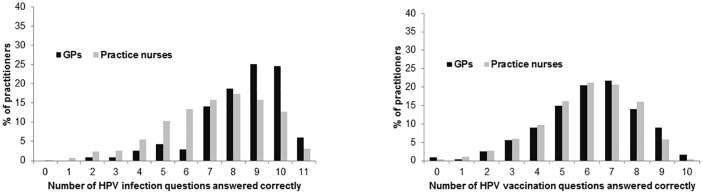
Distribution of HPV infection and vaccination knowledge scores among GPs and practice nurses. **(A)** HPV infection knowledge. (B) HPV vaccination knowledge.

The median number of correctly answered questions was eight. GPs answered more questions correctly than practice nurses (Fig 1(A); GP median = 9; practice nurse median = 7; Wilcoxson rank-sum p<0.001).

#### Factors associated with low knowledge

In the multivariable model, male GPs and practice nurses were significantly more likely than female GPs to have a low HPV infection knowledge score (Table 2). Practitioners who had never taken a cervical cytology test (n = 27), or who took more or fewer of these now than three years ago, had increased likelihood of low knowledge. The likelihood of low knowledge was significantly lower among practitioners whose practice had a public patient list and who worked more hours per week.

**Table 2:** Significant predictors of low HPV knowledge, numbers and percentages with low scores^1^, multivariable odds ratios (OR) with 95% confidence intervals (CI), and p values for point estimates and from likelihood ratio tests (LRT).

		Low score		OR	95%CI		p^2^	LRT p^3^
		No.	%					
**HPV infection**	Practitioner type and gender							
	Female GP	5	4.2	1	-	-	-	
	Male GP	13	16.3	5.95	1.69	20.98	0.006	
	Practice Nurse	93	21.8	5.75	2.13	15.49	0.001	<0.001
	Hours worked per week^4^	-	-	0.95	0.94	0.98	0.000	<0.001
	Practice has public patient list							
	No	68	21.1	1.00	-	-	-	
	Yes	43	14.2	0.46	0.29	0.73	0.001	0.001
	Number of cervical cytology test taken now compared to three years ago							
	I take about the same	4	5.1	1.00	-	-	-	
	I take fewer	12	15.0	5.72	1.48	22.15	0.012	
	I take more	83	18.8	3.71	1.27	10.87	0.017	
	I have never taken a smear	12	48.0	10.56	2.71	41.12	0.001	0.002
**HPV vaccination**	Practitioner type & gender							
	Female GP	8	6.7	1	-	-	-	
	Male GP	26	32.9	3.92	1.34	11.43	0.013	
	Practice Nurse	84	20.4	4.63	1.86	11.52	0.001	<0.001
	Hours worked per week^2^	-	-	0.98	0.95	1.00	0.032	0.030
	Years worked in general practice^3^	-	-	1.05	1.02	1.08	0.003	0.002
	Number of GPs in practice^4^	-	-	0.87	0.77	0.99	0.030	0.023
	Practice specializes in women’s health							
	No	58	28.2	1	-	-	-	
	Yes	60	14.9	0.60	0.39	0.94	0.027	0.028
	Number of cervical cytology test taken now compared to three years ago							
	I take about the same	6	8.0	1	-	-	-	
	I take fewer	23	28.8	4.39	1.43	13.47	0.010	
	I take more	76	17.7	2.11	0.84	5.27	0.110	
	I have never taken a smear	13	52.0	8.51	2.53	28.69	0.001	0.002

^1^ defined as answering ≤5 HPV infection, or ≤4 HPV vaccination, questions correctly

^2^ p value for point estimate (i.e. test of difference of OR from 1)

^3^ p value from likelihood ratio test of contribution of variable to multivariate model.

^4^ OR per unit increase

#### Questions least often answered correctly

Four HPV infection questions (relating to: HPV infection in men increasing risk for anogenital cancers; HPV types associated with cervical cancer and genital warts; location of genital warts and cancer risk; and duration of HPV infection) were not answered correctly by almost half of practitioners (Table 3). More practitioners responded that they were unsure than provided a wrong answer (S1 Table). Two further questions were not answered correctly by at least a third of practitioners (HPV infection causes genital warts: 39% answered incorrectly; clearance of HPV infection: 33% answered incorrectly). More practitioners gave the wrong answer than indicated that they were unsure (S1 Table). For nine questions, female GPs, male GPs and practice nurses differed significantly in the percentage who answered correctly (Table 3).

**Table 3:** Responses to individual HPV infection and vaccination knowledge questions for all practitioners, and by practitioner group, with p values from chi-square tests for association between group and whether response was correct^1^.

Statement	All PCPs		Practitioner Group						
			*Female GPs*		*Male GPs*		*Practice nurses*		*p-value*
	Correct	Incorrect^2^	Correct	Incorrect^2^	Correct	Incorrect^2^	Correct	Incorrect^2^	
**HPV infection**									
a. Genital HPV infection is fairly common in sexually active adults (True)	82.8%	17.2%	91.6%	8.4%	80.4%	19.6%	80.4%	19.6%	p = 0.007
b. A person with genital HPV infection may never show symptoms of infection (True)	91.6%	8.4%	99.3%	0.7%	94.6%	5.4%	88.6%	11.4%	p<0.001
c. Most genital HPV infections may be cleared without medical intervention (True)	67.1%	32.9%	76.2%	23.8%	54.3%	45.7%	66.8%	33.2%	p = 0.002
d. Persistent genital HPV infections in women increase the risk of cervical dysplasia and cervical cancer (True)	93.9%	6.1%	95.8%	4.2%	93.5%	6.5%	93.4%	6.6%	p = 0.569
e. Genital HPV infection in men increases risk of penile and other anogenital cancers (True)	52.8%	47.2%	67.8%	32.2%	72.8%	27.2%	44.0%	56.0%	p<0.001
f. Treatment of cervical dysplasia/cancer always permanently eliminates the causative infection (False)	76.7%	23.3%	86.0%	14.0%	82.6%	17.4%	72.5%	27.5%	p = 0.001
g. Genital HPV infection causes external anogenital warts (True)	61.2%	38.8%	81.8%	18.2%	73.9%	26.1%	52.1%	47.9%	p<0.001
h. Genital HPV types usually associated with external anogenital warts differ from types usually associated with cervical dysplasia and cervical cancer (True)	51.4%	48.6%	64.3%	35.7%	43.5%	56.5%	49.0%	51.0%	p = 0.002
i. External anogenital warts increase risk of cancer at the same site where the warts are located (False)	51.9%	48.1%	56.6%	43.4%	41.3%	58.7%	52.5%	47.5%	p = 0.064
j. Treatment of external anogenital warts always permanently eliminates the causative infection (False)	78.8%	21.2%	92.3%	7.7%	83.7%	16.3%	73.6%	26.4%	p<0.001
k. Available tests and procedures can determine the duration of a patient’s genital HPV infection (False)	53.3%	46.7%	67.8%	32.2%	57.6%	42.4%	47.9%	52.1%	p<0.001
**HPV vaccination**									
a. HPV vaccination gives lifelong protection against cervical cancer (False)	55.2%	44.8%	51.0%	49.0%	38.0%	62.0%	59.0%	41.0%	p<0.001
b. HPV vaccines are live vaccines (False)	69.1%	30.9%	72.7%	27.3%	57.6%	42.4%	69.5%	30.5%	p = 0.028
c. HPV vaccination is generally less effective in sexually active girls/women (True)	65.4%	34.6%	72.0%	28.0%	63.0%	37.0%	62.7%	37.3%	p = 0.114
d. HPV vaccination will prevent >90% of cervical cancers (False)	25.8%	74.2%	28.7%	71.3%	17.4%	82.6%	26.1%	73.9%	p = 0.126
e. HPV vaccines contain no viral DNA and are not infectious or oncogenic (True)	64.6%	35.4%	76.2%	23.8%	60.9%	39.1%	60.6%	39.4%	p = 0.003
f. Available HPV vaccines protect against all of the HPV types that can cause cervical cancer (False)	76.4%	23.6%	81.8%	18.2%	76.1%	23.9%	73.4%	26.6%	p = 0.148
g. HPV vaccination may protect against other types of cancer in addition to cervical cancer (True)	32.6%	67.4%	53.8%	46.2%	50.0%	50.0%	21.8%	78.2%	p<0.001
h. Available HPV vaccines are not licensed for use in adolescent boys in Ireland (True)	49.3%	50.7%	46.9%	53.1%	29.3%	70.7%	53.4%	46.6%	p<0.001
i. Vaccinated females will no longer need to have smears (False)	95.3%	4.7%	95.8%	4.2%	91.3%	8.7%	94.3%	5.7%	p = 0.134
j. All available HPV vaccines protect against genital warts (False)	71.3%	28.7%	80.4%	19.6%	57.6%	42.4%	69.9%	30.1%	p = 0.001

^1^ test of association between practitioner group (female GP/male GP/practice nurse) and whether or not they answered the question correctly (correct/incorrect); whether the statement is true or false is indicted in brackets following each statement

^2^ includes subjects who provided the wrong answer, who indicated that they were unsure, and who declined to answer

### HPV vaccination

#### Knowledge score distribution

For HPV vaccination, of the 687 practitioners who answered at least half the questions, 10 (1.5%) answered zero or one question correctly and six (0.9%) answered all 10 correctly. The median number of questions answered correctly was six. There was no difference between GPs and practice nurses (Wilcoxson rank-sum p = 0.248; Fig 1(B)).

#### Factors associated with low knowledge

For HPV vaccination, the likelihood of a low knowledge score was significantly higher in male GPs and practice nurses than female GPs (Table 2). It was significantly lower with more weekly hours worked and more GPs in the practice, and significantly higher with more years in general practice.

Practitioners whose practice specialised in women’s health were less likely to have low knowledge, while those who had never taken a cervical cytology test, or took fewer now than previously, were more likely to have low knowledge.

#### Questions least often answered correctly

For HPV vaccination, three questions were not answered correctly by more than half of practitioners (Table 3). These related to: percentage of cervical cancers likely to be prevented by vaccination (74% answered incorrectly); whether vaccination offers protection against other cancers (67% answered incorrectly); and whether HPV vaccines are licensed for use in boys (51% answered correctly). For the first, more practitioners answered wrongly than were unsure; for the other two more were unsure than answered wrongly (S1 Table). A further four questions were not answered correctly by between 31% and 45% of respondents. For three of these, more practitioners were unsure than provided the wrong answer (S1 Table). The frequency of correct answers varied significantly between female GPs, male GPs and practice nurses for eight questions (Table 3).

## Discussion

This study investigated HPV knowledge among PCPs in order to inform professional education strategies around HPV. Overall, PCPs correctly answered a median of eight of 11 factual HPV infection questions and six of 10 HPV infection questions. While these figures are not especially low, they conceal important patterns. Notably, practice nurses’ and male GPs’ knowledge levels were significantly lower than female GPs’. Moreover, large proportions of PCPs were wrong or uncertain about several key aspects of HPV-related knowledge.

### HPV knowledge levels over time and internationally

Past studies revealed gaps in PCPs’ HPV knowledge [22–28]. Several of these were conducted before the introduction of HPV vaccination programmes and incorporation of HPV testing into screening, and knowledge may have improved since then. For example, in a 2007 GP survey in Ireland, only 10% of GPs were aware that HPV vaccination may protect against other cancers [32]; this had risen to around 50% in the current survey. However, in the current study, only one in five practice nurses were aware HPV vaccination may protect against other cancers. At the time of our survey, the HPV vaccination programme was in its first year and the national cervical screening programme was completing its first screening round. The suggestion that significant knowledge gaps may remain despite public health developments around HPV is echoed in two recent studies [27,28]. A survey of primary healthcare professionals in Norway, conducted one year after the introduction of HPV vaccination among school-girls, and a survey of primary care practice nurses in England conducted several years after the HPV vaccination programme commenced and use of HPV testing was incorporated into the national screening programme, both revealed limitations in practitioners’ knowledge.

Conclusions of other studies of PCPs’ HPV-related knowledge vary. In one study in Hong Kong, most doctors, nurses and smear-taker trainees had only basic HPV knowledge [33]. In another study, also in Hong Kong, less than half of doctors knew what percentage of cervical cancer is caused by HPV [34]. In contrast, an Australian study reported that GPs have good HPV vaccination knowledge [35]. This indicates the importance of conducting such surveys in settings with different cervical cancer prevention strategies.

### Variations in knowledge between GPs and practice nurses

Our observation that male GPs were more likely to have low HPV knowledge than female GPs is novel. Male GPs may be less interested in the topic of HPV, perceiving it to be a “women’s issue” or related to taking smears which, in Ireland at least, is viewed as a female role [2]. Notably, the only question answered correctly by a greater proportion of male than female GPs concerned the role of HPV in penile and other anogenital cancers. In addition, participating male GPs were sufficiently interested in the topic of cervical cancer prevention to take part. This suggests they may have a better HPV knowledge than the overall population of male GPs [31].

The rationale for the study was theoretically-robust evidence that HPV knowledge is an important predictor of practitioners’ HPV-related clinical behaviours [2] and may influence the quality of advice provided to patients. The concern these results raise is that women who attend male GPs may not receive the highest quality advice. There is a need, therefore, to ensure that any professional development initiatives seek to fully engage male GPs. More widespread recognition of the importance of HPV in cancers at sites other than the female genital tract, and in various benign but common conditions [36,37], may suggest a route through which to engage male GPs with the topic.

Similar to the UK and Australia, practice nurses in Ireland are increasingly delegated the task of taking screening tests [38]. This provides them with opportunities to discuss HPV infection and vaccination with patients. It is worrying, therefore, that practice nurses had lower average HPV infection knowledge scores than GPs and, although median HPV vaccination scores did not differ, for every HPV vaccination question more nurses than female GPs failed to answer correctly. Only two previous studies, the first of 154 practitioners in New Zealand, and the second of 220 practitioners in Norway, appear to have compared GPs’ and primary care nurses’ knowledge [27,39]. In the New Zealand survey, GPs more often correctly answered each of five HPV infection questions. In the Norwegian study, GPs were more knowledgeable about the causal relationship between HPV and cancer and, although nurses knew more about other aspects of HPV than GPs, there were still significant limitations in nurses’ knowledge. These results, and ours, reinforce the need to include practice nurses in any future HPV-related education initiatives.

### Other factors associated with HPV infection and vaccination knowledge

Several factors associated with HPV infection knowledge were also related to HPV vaccination knowledge. This was unsurprising given that infection and vaccination knowledge levels were significantly correlated.

Several “practice”-related factors were significantly associated with low scores, especially for HPV vaccination. GPs’ factual medical knowledge may decline with age and full-time practitioners tend to have higher knowledge scores than part-time practitioners [40]. The observed associations between low HPV knowledge and working more years in general practice and working fewer hours per week are probably due to this. Older practitioners, who trained when there was less emphasis on continuing medical education (CME), may be less likely to keep up-to-date with medical developments, and those who work fewer hours may have less opportunity to do so. Working in a smaller practice was significantly associated with low HPV vaccination knowledge. Practitioners in smaller practices may be more isolated from recent developments or less likely to attend CME or other training events. They may also receive fewer visits from representatives of the HPV vaccine manufacturers. The association between low knowledge and never having taken a smear was unsurprising. Practitioners who take smears for the screening programme are encouraged to attend training and information sessions provided by CervicalCheck, some of which cover HPV.

### Specific limitations in knowledge

We assessed HPV infection knowledge using questions developed in Jain et al. [22]. For most questions, a higher percentage of the 368 US family physicians in that study (who were surveyed in 2004) provided correct answers than the PCPs in our study. Two exceptions were the questions on whether infection may clear without treatment and whether genital warts increase cancer risk at the site of the wart; both questions were answered correctly by more PCPs in the current study but high proportions still failed to provide a correct answer (33% and 48% respectively). Moreover, around half of practitioners in both settings were unable to correctly answer a question about whether the same HPV types are associated with genital warts and cervical dysplasia. A subsequent US study reported low awareness among physicians that HPV vaccination may prevent vaginal, vulvar and anal cancer [41]. Similarly, in our study, two-thirds of practitioners were unable to correctly answer a question about this. Less than 20% of healthcare providers in China knew that sexually naive women are the most appropriate population for HPV vaccination [42]. The percentage of correct answers to a similar question in the current study was higher, but one-third of practitioners failed to answer correctly. The similarities in the findings of these studies suggest that practitioners in many settings may be unclear about these aspects of HPV.

### Implications

The observed gaps in HPV knowledge of PCPs are particularly concerning given the recommended change to primary HPV-based screening in Ireland [43] (and similar changes underway or imminent elsewhere). There is an urgent need to develop professional education initiatives for PCPs to ensure that they are well informed and that women have access to accurate and high-quality HPV-related information and advice. If such initiatives are not provided, it is possible that screening uptake might fall (because of the link between practitioners’ knowledge and HPV-related behaviours [2,12]) or the psychological burden on women of having a positive screening test might rise (since women consider PCPs trusted sources of HPV information [19–21]). The study findings may be helpful in terms of targeting such initiatives (e.g. to practitioner groups with lower knowledge) and developing content (e.g. focusing on areas of HPV were knowledge gaps are greatest). Since PCPs’ have many competing priorities, educational resources might be provided in different formats so that PCPs can access these in a format, and at times, that suits them. In terms of incentives, training and education events could have CME accreditation. It may also be worth considering whether completion of, for example, a HPV education course should a pre-condition of being approved as a screening provider by the screening programme.

From a research perspective, although knowledge *per se* is a key determinant of physicians undertaking cervical cancer prevention behaviours [2,12], it remains unclear which specific aspects of HPV knowledge are related to provision of appropriate HPV information or advice, HPV tests or other HPV-related clinical behaviours. To underpin development of effective professional educational initiatives, research is needed to better understand which individual aspects of knowledge drive specific behaviours.

### Strengths and limitations

Low response rates by PCPs to mailed surveys are common, and our response rate (40%) is consistent with those typically reported [44]. We do not know why PCPs did not take part and can only speculate that reasons may include lack of interest in the topic, other priorities, or lack of time. As with any survey, it is possible that participants’ knowledge levels differ from those of the populations from which they were drawn. Participating GPs’ characteristics were similar to those of all GPs in the Irish Medical Directory, with the exception of gender; female GPs were over-represented among survey participants. Other than knowing that they were all female, we had no data on characteristics of the nurses comprising the sampling frames. The data collection was in 2010, and that is a limitation. We cannot be certain about current knowledge levels. If the survey was repeated now knowledge levels may be higher as practitioners have become more familiar with these screening and vaccination programmes. Alternatively, they may have fallen; HPV was considered a “hot topic” at the time of the survey and this may have placed it at the forefront of practitioners’ minds.

### Conclusions

There are important limitations in PCPs’ HPV infection and vaccination knowledge particularly among male GPs and practice nurses. Characteristics of practitioners most likely to need support, and specific aspects of HPV-related knowledge about which practitioners are most often uncertain or incorrect, have been identified. The results can inform development of professional education initiatives to ensure that women have access to uniformly high-quality HPV information and advice.

## Supporting information

S1 Table Responses to individual HPV infection and vaccination knowledge statements for all practitioners, according to whether they provided correct answers, provided wrong answers, indicated that they were unsure or did not respond^1^. (DOCX)
