# Supplementary material for: Which primary care practitioners have poor human papillomavirus (HPV) knowledge? A step towards informing the development of professional education initiatives
Source: PLoS One. 2018 Dec 13;13(12):e0208482. doi: 10.1371/journal.pone.0208482 (PMC6292662; doi:10.1371/journal.pone.0208482)
Supplement: S1 Table — (DOCX) [file pone.0208482.s001.docx]

| **Statement** | **Correct** | **Wrong** | **Unsure** | **No response** |
| --- | --- | --- | --- | --- |
| **HPV infection** |  |  |  |  |
| - Persistent genital HPV infections in women increase the risk of cervical dysplasia and cervical cancer | 93.9% | 17.0% | 22.0% | 3.0% |
| - A person with genital HPV infection may never show symptoms of infection | 91.6% | 4.1% | 4.1% | 0.3% |
| - Genital HPV infection is fairly common in sexually active adults | 82.8% | 10.6% | 5.8% | 0.9% |
| - Treatment of external anogenital warts always permanently eliminates the causative infection | 78.8% | 4.6% | 15.7% | 0.9% |
| - Treatment of cervical dysplasia/cancer always permanently eliminates the causative infection | 76.7% | 3.2% | 19.7% | 0.4% |
| - Most genital HPV infections may be cleared without medical intervention | 67.1% | 19.9% | 12.0% | 1.0% |
| - Genital HPV infection causes external anogenital warts | 61.2% | 23.0% | 14.9% | 0.9% |
| - Available tests and procedures can determine the duration of a patient’s genital HPV infection | 53.3% | 2.8% | 43.0% | 0.9% |
| - Genital HPV infection in men increases risk of penile and other anogenital cancers | 52.8% | 10.4% | 35.4% | 1.9% |
| - External anogenital warts increase risk of cancer at the same site where the warts are locates | 51.9% | 14.5% | 32.5% | 1.2% |
| - Genital HPV types usually associated with external anogenital warts differ from types usually associated with cervical dysplasia and cervical cancer. | 51.4% | 19.1% | 28.1% | 1.3% |
| **HPV vaccination** |  |  |  |  |
| - Vaccinated females will no longer need to have smears | 95.3% | 0.9% | 3.5% | 0.3% |
| - Available HPV vaccines protect against all of the HPV types that can cause cervical cancer | 76.4% | 13.0% | 9.7% | 0.9% |
| - All available HPV vaccines protect against genital warts | 71.3% | 11.8% | 15.6% | 1.3% |
| - HPV vaccines are live vaccines | 69.1% | 7.7% | 20.5% | 2.6% |
| - HPV vaccination is generally less effective in sexually active girls/women | 65.4% | 22.3% | 11.2% | 1.2% |
| - HPV vaccines contain no viral DNA and are not infectious or oncogenic | 64.6% | 3.2% | 30.7% | 1.5% |
| - HPV vaccination gives lifelong protection against cervical cancer | 55.2% | 18.6% | 25.8% | 0.4% |
| - Available HPV vaccines are not licensed for use in adolescent boys in Ireland | 49.3% | 11.1% | 38.4% | 1.2% |
| - HPV vaccination may protect against other types of cancer in addition to cervical cancer | 32.6% | 40.3% | 26.1% | 1.0% |
| - HPV vaccination will prevent >90% of cervical cancers | 25.8% | 58.5% | 14.9% | 0.9% |

^1^ in each section, questions are ordered by decreasing % answered correctly
